# Domestic violence in the perceptions of university students in Poland and Belarus

**DOI:** 10.5249/jivr.vo112i2.1504

**Published:** 2020-07

**Authors:** Marta Giezek, Andrei Shpakou, Paulina Zabielska, Beata Karakiewicz

**Affiliations:** ^*a*^ Subdepartment of Social Medicine and Public Health, Department of Social Medicine, Pomeranian Medical University in Szczecin, Poland.; ^*b*^ Yanka Kupala State University of Grodno, Belarus.

**Keywords:** Domestic violence, Europe, Students

## Abstract

**Background:**

Domestic violence is a social phenomenon where a family member, husband, wife or another cohabiting person in the household tries to dominate, physically or mentally, the other partner, children, parents, grandparents, in-laws, etc., using their physical advantage, threats, blackmail, with the intention of harm. The aim of the study was find the perceptions of university students regarding the phenomenon of violence and to show similarities and differences in this respect between students from Poland and Belarus (PL and BY).

**Methods:**

A total of 482 persons took part in the study, including 251 students from Szczecin (Poland) and 231 students from Grodno (Belarus). The method was a diagnostic survey using the authors’ original questionnaire. The questionnaire consisted of two parts: the first, containing 6 demographic questions, and the second containing 25 mainly closed-ended questions.

**Results:**

In the perceptions of the respondents from Poland and Belarus, women are statistically more likely to experience violence, and men are more likely to use it. The responses of students from Poland and Belarus show statistical connections regarding such behaviors as a single spank, shouting, refusing to talk – recognizing them mostly as a form of violence. The respondents from both countries also show a convergent position concerning quarrelling and forcing a person to drink alcohol or smoke cigarettes, recognizing them as violent behavior. Significant differences can also be observed with regard to such behaviors as throwing objects, name-calling and mocking, which do not indicate violence in the opinions of the majority of students from Belarus, in contrast to the Polish students, who perceive these behaviors as violent.

**Conclusions:**

Students from both countries acknowledge that the concealment of domestic violence by victims mainly results from fear of worsening their already difficult situation as well as from fear of retaliation by the perpetrator.

## Introduction

Family violence is a widespread phenomenon affecting all social groups, but the world literature on the subject indicates that women are more vulnerable to it – as was confirmed by the perceptions of university students from Poland and Belarus. Studies conducted by the World Health Organization show that 10-69% of women worldwide have been physically attacked by their partners at least once in their lives. In Australia, Canada, Israel, South Africa and the United States, between 40% and 70% of all murdered women died at the hands of their partners.^1^ However, domestic violence does not only affect women, as indicated by the TNS OBOP study of 2010, men constitute approximately 39% of the total number of victims of domestic violence,^2^ which was confirmed by the perception of the surveyed students. 

In 1988, the Institute of Medicine (IOM) released a Domestic violence has the following characteristics: 

1. It is intentional – deliberate and consciously aimed at subjugating the victim.

2. There is an imbalance – in the relationship, one of the partners has an advantage over the other. 

3. It violates personal rights – the perpetrator uses their advantage to violate fundamental rights of the victim (e.g. the right to physical integrity, dignity, respect, etc.).

4. It causes pain and suffering – the perpetrator threatens the victim’s life and health. 

Pursuant to the Act on counteracting domestic violence of 29 July 2005, domestic violence involves a single or multiple deliberate acts or omissions infringing the rights of family members, especially exposing them to the risk of loss of life, health, violating their dignity, physical integrity, freedom, including sexual freedom, causing damage to their physical or mental well-being, as well as causing suffering and non-material losses in those affected by violence.^3^ The Convention on preventing and combating violence against women and domestic violence in its article 3 b) defines domestic violence as all acts of physical, sexual, psychological or economic violence that occur within the family or domestic unit or between former or current spouses or partners, whether or not the perpetrator shares or has shared the same residence with the victim.^4^ Domestic violence may be passive – manifesting itself as non-interaction, or active, taking a number of different forms: physical – with assaults, psychological, sexual, economic as well as neglect – most commonly aimed at children and the elderly or disabled members of the family.^5^ There is no single cause of violence. The causes most frequently mentioned in the literature on the subject include unconscious copying of bad cultural and social role models, insufficient social and emotional competence to cope with difficult situations, and the substitution of violence and aggression for lack of self-esteem.^6,7^

There are several concepts of violence in the literature on the subject, but domestic violence is addressed by Albert Cohen’s subcultural theory of deviance, developed in the 1950s. It claimed that aggression and violence are characteristic of the lower social classes and observed in dysfunctional families. This image of a family affected by violence was common even less than 20 years ago. However, this theory failed to explain why violence also afflicted the so-called “good homes”, which gave rise to theories on white-collar violence. These days, our perceptions of domestic violence are closer to the neutralization theory described by David Matza and Gresham Sykes, where the perpetrator is seen as the person administering justice, and the victim is treated as if they deserve the punishment.^8^

Domestic violence is a worldwide phenomenon which has not only personal but also social nature. It results from an interaction of cultural, social, legal, health, psychological and economic factors – therefore there is no single reason for domestic violence.^6^ In many countries, including Poland and Belarus, the use of domestic violence has been condoned for centuries, and although legal regulations began to be introduced in order to protect family members against violence, the deep-rooted myths and stereotypes concerning family roles, the position of women, the dominant role of husbands and fathers, as well as the status of children and the elderly make the phenomenon of domestic violence extremely difficult to eradicate. ^9,10^ In this article, we compare two countries, Poland and Belarus, which historically did not differ much in terms of mentality. In the past 50 years Poland has made great steps towards democracy, while Belarus stuck to its roots, maintaining views on domestic violence based on the principles of the Soviet Union, as an excellent example of a post-Soviet country. The right to protection against domestic violence is one of the obligations of the state, and failure to provide it may be considered a violation of human rights.^11^ However, both in Poland and Belarus, just like in many other countries, the effectiveness of actions taken is limited by victims not reporting violence, which simultaneously hinders the estimation of the scale and character of this phenomenon.^12,13^

Domestic violence can emerge and develop in ways which are difficult for victims to define unambiguously, e.g. through small acts of control, humiliation or isolation performed in ways that are not expressive enough to cause alarm.^14^ Victims also frequently believe that the abuse they experience is their personal problem, which prevents their search for support and protection in relevant institutions.^15^ The functioning of families based on an unequal position of power and control, as one of the criteria distinguishing domestic violence from other forms of action or inaction, makes victims and witnesses lose vigilance and, as a result, fail to notice the manifestations of violence, especially if there are no acts of physical violence.^16^ Another factor exerting an influence on the perception of the phenomenon of violence, especially by young people, is easy access to the Internet, as well as online anonymity. For young people, the boundary between the virtual world and the real world is often blurred.^17^ Due to the scale of the phenomenon, domestic violence prevention has been one of the key topics in social policy in recent years. This dysfunctional phenomenon is analysed in terms of causes, types and forms, mechanisms, predisposing factors, social groups, consequences, as well as the possibilities of provision of assistance and therapy for both victims and perpetrators. It is recommended that all actions aimed at eradication of domestic violence should have an interdisciplinary character.^3^ Poland has a coordinated help system in place for people suffering domestic violence, developed at the ministerial level in the form of the Blue Card procedure, covering all of the measures undertaken and followed by the organizational units of social services, local commissions for resolving alcohol-related problems, police, education and healthcare system, whenever there are reasonable grounds to suspect violence in the family.^18^

In Belarus, there is no Blue Card procedure or a similar one – the main document which includes the concept of “domestic violence” is the Act on the Fundamentals of Crime Prevention. In 2002, the first bill on preventing and combating domestic violence was drafted in Belarus, but it was not passed.^19^ The aim of the study was to find the perceptions of domestic violence by university students in Poland and Belarus, with special attention being paid to similarities and differences between the students from both countries in understanding the causes of violence, its types and forms, and the reasons why it is not reported. 

## Material and Method

A total of 482 persons took part in the study, including 251 students (196 women and 55 men) of the Department of Health Sciences of the Pomeranian Medical University in Szczecin (Poland) and 231 students (155 women and 76 men) of the Yanka Kupala State University of Grodno (Belarus). The study included 81,1 % of all students in Grodno, Belarus and 15,5% in Szczecin, Poland. Among those surveyed, women formed the majority both in Grodno and Szczecin. This reflects the demographic trend observed in both countries, whereby the student population is predominantly female. The choice of the study population was fully conscious and justified. Third-level students, by definition, are supposed to make upstanding citizens of society in the future, models to be followed. They should therefore represent a higher level of knowledge and personal culture, and also demonstrate higher awareness on issues of culture, health and opposing violence. Students, more than others, should be expected to be experts and leaders of public health-promoting activity.^20^ Detailed socio-demographic data are shown in Table 1.

**Table 1:** Characteristics of respondents.

Variable		POLAND 251 (100%)	BELARUS 231 (100%)
**Sex**	Women	196 (78.1%)	155 (67.1%)
	Men	55 (21.9%)	76 (32.9%)
**Age**	19-25	211 (84.1%)	185 (80.1%)
	26-29	40 (15.9%)	46 (19.9%)
**Place of residence**	Rural area	47 (18.7%)	47 (20.3%)
	City>50.000	59 (23.5%)	27 (11.7%)
	City 50.000-300.000	21 (8.4%)	46 (19.9%)
	City<300.000	124 (49.4%)	111 (48.1%)

The method was a diagnostic survey using the authors’ original questionnaire. The questionnaire consisted of two parts: the first, containing 6 demographic questions, and the second containing 25 mainly closed-ended questions. The survey questionnaire used in the study was not validated statistically. The questionnaire for students from Grodno was translated into Belarusian by means of back translation. To guarantee reliability and full compatibility of questionnaire content in both languages, a double blind translation was used. The tool was provided for students to complete in an online version. 

The calculations were made by means of the R statistical calculation environment and Microsoft Excel spreadsheet, using the standard functions of the program. Due to the fact that the distribution was different than normal (distributions of variables were tested with the Shapiro-Wilk test), descriptive statistics included the characteristics of variables using the mean (M), standard deviation, median (Me), maximum and minimum (Max. - Min.) values and percentile values (25% -75% quartiles). The nonparametric Mann-Whitney U test for medians was used to compare differences between the groups. The distribution of qualitative variables was shown by means of frequency and proportion with 95% confidence intervals. Statistical comparisons were completed using Chi-square test of independence. Significant differences were defined as p<0.05. 

## Results

For the purposes of this study, 18 factors associated with the increased probability of domestic violence were formulated. Their distribution, depending on the country of study, is presented in Table 2. Only “Yes” or “No” responses were counted.

**Table 2:** Factors associated with higher probability of domestic violence in the perceptions of Polish and Belarusian students.

Cause of violence	Number of respondents without the “no answer” option, n		BY		Responses	PL		Pearson Chi-square, (P)*
	BY	PL	Yes	No		Yes	No	
Addictions	213	242	94.4(91.3-97.5)	5.6(2.5-8.7)		97.9(96.1-97.7)	2.1(0.3-3.9)	4.0.(<0.05)
Mental disorders	206	223	87.9(8.34-92.3)	12.1(7.7-16.6)		94.2(91.1-97.3)	5.8(2.8-8.9)	5.3.(<0.02)
No positive role models in the family	184	224	68.5(61.8-75.2)	31.5(24.8-38.2)		95.1(92.3-97.9)	4.9(2.1-7.7)	50.9.(<0.01)
Bad company	195	223	74.9(68.8-81.0)	25.1(19.0-31.2)		94.6(91.7-97.6)	5.4(2.4-8.3)	32.5(<0.01)
Lack of parents’ time for children	177	189	53.7(46.3-61.0)	46.3(39.0-53.7)		70.9(64.4-77.4)	29.1(22.6-35.6)	11.6(<0.01)
Early sexual initiation	173	166	35.3(28.1-42.4)	64.7(57.6-71.9)		34.9(27.7-42.2)	65.1(57.8-72.3)	0.1(>0.1)
Poverty	185	195	51.9(44.7-59.1)	89 (48.1(40.9-55.3)		69.7(63.3-76.2)	30.3(23.8-36.7)	12.7(<0.01)
Unemployment	183	202	53.4(50.2-64.6)	42.6(35.5-49.8)		79.2(73.6-84.8)	20.8(15.2-26.4)	21.3(p<0.01)
Homelessness	170	174	52.9(45.4-60.4)	47.1(39.6-54.6)		56.3(49.0-63.7)	43.7(36.3-51.1)	0.4(>0.1)
Long-term illness and need for care	171	185	25.1(18.7-31.7)	74.9(68.4-81.4)		44.3(37.2-51.5)	55.7(48.5-62.8)	14.3(<0.01)
Disability	178	189	34.3(27.3-41.2)	65.7(58.8-72.7)		36.5(29.7-43.4)	63.5(56.6-70.4)	0.2(p>0.05)
Watching violent movies	179	180	58.1(50.9-65.3)	41.9(34.7-49.1)		60.0(52.8-67.2)	40.0(32.8-41.2)	0.1(>0.1)
Computer games	187	187	64.2(57.3-71.0)	35.8(29.0-42.7)		65.2(58.4-72.1)	34.8(27.9-41.6)	0.04(>0.1)
Frustration	166	232	44.6(37.0-57.1)	55.4(47.9-63.0)		93.1(89.8-96.4)	6.9(3.6-10.2)	115.2(<0.01)
Complexes	174	209	39.7 (32.4-46.9)	60.3 (53.1-67.6)		82.3(77.1-87.5)	17.7(12.5-22.9)	74.0 (p<0.01)
Lack of self-contained accommodation	179	166	29.6(22.9-36.3)	70.4(63.7-77.1)		38.5(31.2-46.0)	61.5(54.1-68.9)	3.1(<0.08)
Jealousy	183	209	59.6(52.5-66.7)	40.4(33.3-47.6)		83.7(78.7-88.7)	16.3(11.3-21.3)	28.6(<0.01)
Stereotypes (e.g. you should love, respect and obey)	173	182	57.8(50.4-65.2)	42.2(34.8-49.6)		71.4(64.9-78.0)	28.6(22.0-36.1)	7.2(<0.01)

* Results of Chi-square test

The answers provided by the respondents show the unanimity of Polish and Belarusian students regarding factors stimulating the occurrence of domestic violence such as early sexual initiation, homelessness, disability, watching pornographic films and playing computer games. In the case of factors causing violence such as frustration and complexes, a significant statistical difference can be observed between respondents from the two countries. In both cases, students from Poland believe that this is a significant cause of violence (frustration: yes – 216 (93.1%), no – 16 (6.9%), complexes: yes – 172 (82.3%), no – 37 (17.7%), whereas students from Belarus do not have such a strong position (frustration: yes – 74 (44.6%), no – 92 (55.4%), complexes: yes – 69 (39.7 %), no – 105 (60.3%). 

Another variable subjected to the respondents’ perceptions was the determination of the profile of persons involved in domestic violence depending on gender. Students expressed their opinions by assigning a point value from 1 to 5, with 5 being the most significant predictor and 1 having the least importance, as is presented in Table 3.

**Table 3:** Gender and profile of persons involved in violence in the perceptions of Polish and Belarusian students.

Gender	Profile	Group	Mean±SD	Minimum -Maximum	Q25	Median	Q75	Mann-Whitney U Test
Female	victim	BY	3.72±1.78		2.00	5.00	5.00	0.65
		PL	3.78±1.88		2.00	5.00	5.00	
	perpetrator	BY	2.24±1.98		0.00	2.00	4.00	0.20
		PL	1.99±1.75	0-5.00	0.00	2.00	4.00	
Male	victim	BY	2.05±1.74		1.00	1.00	4.00	0.33
		PL	1.88±1.69		0.00	2.00	4.00	
	perpetrator	BY	3.25±1.99		1.00	4.00	5.00	0.001
		PL	3.66±1.86		2.00	5.00	5.00	

The results of the study confirm literature reports on the subject of domestic violence, according to which violence against women is a common phenomenon occurring in every region of the world. Every third woman in the world experiences physical or sexual violence by the man with whom she has an intimate relationship.^21,22^ In the perceptions of the respondents from Poland and Belarus, women are statistically more likely to experience violence, and men are more likely to use it (Table 4).

**Table 4:** Different types of violence in the perceptions of Polish and Belarusian students.

Behavior	BY (n=231)		Responses	PL (n=251)		Pearson Chi-square, (P)*
	Yes, this is violence	This is not violence		Yes, this is violence	This is not violence	
			**PHYSICAL VIOLENCE**			
Single spank	80.5 (75.4-85.6)	19.5 (14.4-24.6)	****	80.9 (76.0-85.8)	19.1 (14.3-24.0)	0.1 (>0.9)
Hair pulling	11.3 (7.2-15.3)	88.7 (84.7-92.8)	****	15.1 (10.7-19.6)	84.9 (80.4-82.3)	1.6 (>0.2)
Beating with a belt	44.2 (37.8-50.6)	55.8 (49.4-62.2)	****	9.6 (5.9-13.2)	90.4 (86.8-94.1)	74.5 (<0.001)
Slapping	45.9 (39.5-52.3)	54.1 (47.7-60.5)	****	10.0 (6.3-13.7)	90.0 (86.3-93.7)	78.5 (<0.0015
Restriction of food	36.8 (30.6-43.0)	63.2 (57.0-69.4)	****	17.1 (12.5-21.8)	82.9 (78.2-87.5)	23.9 (<0.001
			**PSYCHOLOGICAL ABUSE**			
Name-calling	60.6 (54.3-66.9)	39.4 (33.1-45.7)	****	17.5 (12.8-22.2)	82.5(77.8-87.2)	94.6 (<0.001
Mocking	60.6 (54.3-66.9)	39.4 (33.1-45.7)	****	17.1 (12.5-21.8)	82.9 (78.2-85.7)	96.5 (<0.001
Enforcing obedience	52.8 (46.4-59.3)	47.2 (40.8-53.6)	****	16.7 (12.1-21.4)	83.3 (78.7-87.9)	69.8 (<0.001
Refusing to talk	83.1 (78.3-88.0)	16.9 (12.1-21.7)	****	79.7 (74.7-84.7)	20.3 (15.3-25.3)	0.9 (>0.3)
Restriction of social contacts	60.6 (54.3-66.9)	39.4 (33.1-45.7)	****	35.1 (29.2-41.0)	64.9 (59.0-70.8)	31.5 (<0.001
Intimidation	33.3 (27.3-39.4)	66.7 (60.6-72.8)	****	7.6 (4.3-10.8)	92.4 (89.2-95.7)	50.1 (<0.001)
			**ECONOMIC ABUSE**			
Taking the victim’s money	82.7 (77.8-87.6)	17.3 (12.4-22.2)	****	43.4 (37.3-49.6)	56.6 (50.4-62.7)	78.9 (<0.001
Preventing the victim from going to work	67.5(61.5-73.6)	32.5 (26.4-38.5)	****	27.9 (22.3-33.4)	72.1 (66.6-77.7)	75.9 (<0.001
			**SEXUAL ABUSE**			
Forcing sex on the partner	12.6 (8.3-16.8)	87.4 (83.2-91.7)	****	5.2 (2.4-7.9)	94.8 (92.1-97.6)	8.2 (<0.01)
Forcing the partner to watch films (pornography)	33.8 (27.7-39.9)	66.3 (60.1-72.3)	****	19.5 (14.6-24.4)	80.5 (75.6-85.4)	12.6 (<0.001)
			**OTHER TYPES OF ABUSE **			
Forcing the victim to drink alcohol or smoke cigarettes	22.9m(17.5-28.4)	77.1 (71.6-82.5)	****	17.5 (12.8-22.2)	82.5 (77.8-87.2)	2.2 (>0.1)
Shouting	67.1 (61.0-73.2)	32.9 (26.8-39.0)	****	61.4 (55.3-67.4)	38.6 (32.6-44.7)	1.7 (>0.1)
Quarrelling	78.8 (73.5-84.1)	21.2 (15.9-26.5)	****	73.7(68.3-79.2)	26.3(20.8-31.7)	1.7 (>0.1)
Throwing things	67.5 (61.5-73.6)	32.5 (26.4-38.5)	****	24.3 (19.0-29.6)	75.7 (70.4-81.0)	90.8 (<0.001)
Locking the victim in their room	52.0 (45.5-58.4)	48.0 (41.6-54.5)	****	31.9 (26.1-37.6)	68.1 (62.4-73.9)	20.0 (<0.001)

* Results of Chi-square test.

The responses of students from Poland and Belarus show statistical connections regarding such behaviors as a single spank, shouting, refusing to talk – recognizing them mostly as a form of violence. The respondents from both countries also show a convergent position concerning quarrelling and forcing a person to drink alcohol or smoke cigarettes, recognizing them as violent behavior. The lack of connection in the assessment of violent behavior between Polish and Belarusian young people is visible in the response regarding “slapping”, where the result of Pearson’s χ2 test is 78.5. Significant differences can also be observed with regard to such behaviors as throwing objects, name-calling and mocking, which do not indicate violence in the opinions of the majority of students from Belarus, in contrast to the Polish students, who perceive these behaviors as violent. 

In the case of domestic violence, it is the family home where acts of aggression take place, and are often concealed from those around. They come to light only in the aftermath of tragedy. Acts of aggression mainly affect physically weaker individuals – most often women and children. The feeling of helplessness, living in constant anxiety as well as socio-cultural, emotional and religious factors make the family member who is a victim of violence conceal this fact, which meets the perpetrator’s expectations.^23,24^ The questionnaire included the question, “Why do victims of domestic violence conceal it?” The students were asked to select the answers which they considered relevant and grade them from 1 to 5, with 5 being the most significant predictor and 1 the least significant predictor (Table 5).

**Table 5:** Reasons for concealment of violence.

Why do victims of violence conceal it?	Group	Gender	Mean	±SD	Median	Mann-Whitney U Test, Z, p
**They are ashamed**	BY (n=153)	M (n=54)	4.07	1.45	5.0	-0.5, >0.05
		F (n=99)	4.30	1.25	5.0	
		**Total**	4.22	1.32	5.00	-0.8, >0.1
			4.40	1.10	5.00	
	PL (n=156)	M (n=23)	4.61	0.50	5.0	-0.3, >0.7
		F (n=133)	4.37	1.17	5.0	
**They fear that their situation will become even worse if they tell someone**	BY (n=136)	M (n=51)	3.59	1.47	4.0	-2.1, <0.05
		F (n=85)	4.08	1.36	5.0	
		**Total**	3.90	1.42	4.0	-4.6, <0.01
			4.60	0.95	5.0	
	PL (n=166)	M (n=23)	4.91	0.29	5.0	1.3, >0.1
		F (n=143)	4.55	1.01	5.0	
**They love the perpetrator**	BY (n=109)	M (n=40)	3.18	1.66	4.0	0.8, >0.3
		F (n=69)	2.93	1.64	2.0	
		**Total**	3.02	1.64	2.0	-1.1, >0.1
			3.29	1.65	4.0	
	PL (n=118)	M (n=25)	2.76	1.76	2.0	-1.9, =0.05
		F (n=93)	3.43	1.60	4.0	
**They believe that the perpetrator will change**	BY (n=127)	M (n=48)	3.15	1.61	2.0	-0.2, >0.8
		F (n=79)	3.33	1.65	4.0	
		**Total**	3.26	1.63	4.0	-1.4, >0.1
			3.60	1.52	4.0	
	PL (n=132)	M (n=21)	3.67	1.39	4.0	-0.05, >0.9
		F (n=111)	3.59	1.55	4.0	
**They protect the perpetrator from punishment**	BY (n=135)	M (n=56)	2.61	1.58	2.0	0.4, >0.7
		F (n=79)	2.52	1.58	2.0	
		**Total**	2.56	1.58	2.0	-0.1, >0.5
			2.61	1.64	2.0	
	PL (n=119)	M (n=21)	2.05	1.43	1.0	-1.6, >0.1
		F (n=98)	2.72	1.66	2.0	
**They believe that this is their fate and nothing can be done about it**	BY (n=121)	M (n=45)	2.93	1.71	2.0	1.4, >0.1
		F (n=76)	2.47	1.63	2.0	
		**Total**	2.64	1.67	2.0	-0.7, >0.1
			2.85	1.68	2.0	
	PL (n=100)	M (n=21)	2.52	1.75	1.0	-1.4, >0.1
		F (n=79)	2.94	1.67	2.0	
**“What God has joined together…”**	BY (n=101)	M (n=37)	2.97	1.64	2.0	0.3, >0.7
		F (n=64)	2.91	1.65	2.0	
		**Total**	2.93	1.64	2.0	0.5, >0.5
			2.80	1.74	2.0	
	PL (n=92)	M (n=24)	2.21	1.69	1.0	-2.1, <0.05
		F (n=68)	3.01	1.71	2.0	
**They fear they will not cope with life**	BY (n=121)	M (n=45)	3.27	1.71	4.0	0.4, >0.7
		F (n=76)	3.09	1.60	3.0	
		**Total**	3.16	1.64	4.00	-1.7, >0.05
			3.54	1.59	4.00	
	PL (n=132)	M (n=26)	3.08	1.57	4.0	-1.8, >0.05
		F (n=106)	3.65	1.58	4.0	
**They blame themselves**	BY (n=117)	M (n=36)	3.17	1.75	3.0	0.9, >0.3
		F (n=81)	2.94	1.70	2.0	
		**Total**	3.01	1.71	2.00	-0.6, >0.5
			3.16	1.68	4.00	
	PL (n=142)	M (n=24)	2.79	1.72	2.0	-1.0, >0.3
		F (n=118)	3.24	1.67	4.0	
**They have nowhere to go**	BY (n=120)	M (n=41)	3.20	1.69	4.0	-1.1, >0.1
		F (n=79)	3.56	1.65	4.0	
		**Total**	3.43	1.67	4.0	-0.7, >0.5
			3.70	1.55	4.0	
	PL (n=147)	M (n=23)	3.48	1.73	4.0	-0.7, >0.5
		F (n=124)	3.74	1.52	4.0	

In the survey of the students’ perceptions of reasons for concealment of violence, the respondents were divided according to gender. It can be concluded that in the case of students from Poland or Belarus gender was not significant in determining the reasons for victims concealing the experience of violence. The statistically dominant answer in this respect was: “They are afraid that their situation will become even worse if they tell someone”, which confirms the stereotypical view of violence as a secret, the disclosure of which could have unpleasant consequences.

## Discussion

The Belarussian Constitution guarantees all citizens equality before the law, and it guarantees women equal access to education, employment, as well as socio-economic, cultural and other spheres of activity.^25^ The Polish Constitution also guarantees Polish citizens equality before the law.^26^ In most countries of the world there are acts of law condemning family violence, but dealing with the problem in many cases depends not so much on the effectiveness of the procedures or the operation of the services, but on the reporting of the crime by victims themselves or by witnesses of domestic violence. The true scale of the incidence of domestic violence is not known in any country in the world as many cases remain unregistered and, unfortunately, are still often considered a private issue. Only slightly over a quarter of the victims are provided with assistance. Most often, victims use the assistance of the police (14%), which happens much more frequently in the case of women than men (32% and 17%, respectively).^2^ In the respondents’ perceptions, the concealment of violence by victims is caused mainly by fear of further worsening of their situation, but also for religious, cultural, economic, social and emotional reasons, which is consistent with the literature on the subject. ^27^

The scale of the phenomenon of domestic violence is indicated by statistics conducted mainly by the police, based on the use of specific tools. In Poland, a tool of this kind is the number of “Blue Cards” procedures initiated.^18^ A total of 73,153 “Blue Cards” were established by the police in 2018, including 59,829 initiating the procedure and 13,324 concerning subsequent incidents during the procedure.^27^

In January 2004, Belarus presented data from the Women’s Convention, during which government representatives pointed to violence against women as one of the major challenges which need to be met in order to achieve gender equality, while declaring that 30% of Belarusian women were victims of domestic violence and 12% were victims of sexual abuse at workplace.^28^ Belarus has a high divorce rate: only in 2003 there were 31,700 divorces out of 69,900 marriages contracted, and in 64% of the cases the reason for divorce was beating and alcohol.^29^ In 2001-2002, UNIFEM examined the level of public awareness of domestic violence and sexual abuse at workplace, as well as their coverage in the media. The research was conducted in Azerbaijan, Belarus, Kazakhstan, Kyrgyzstan, Lithuania, Moldova, Russia, Tajikistan and Uzbekistan. Most of the populations in all the countries under study accepted relationships in which violence occurred. Violence as a normal element of a male-female intimate relationship was accepted to a lesser extent in Belarus than in Azerbaijan, Tajikistan and Uzbekistan. ^30^ Belarusian criminal procedures require victims to report crime, hence the fear of retaliation by the partners, self-blame, fear of bringing shame to the family, low self-esteem and economic insecurity are frequent reasons for victims refraining from reporting domestic violence or withdrawing their statements in Belarus, ^30,31^ which was confirmed by the results of this study. 

In 2018, the Belarusian Ministry of Interior prepared the concept for a new law on combating domestic violence, which extended the notion of “domestic violence” and included, in addition to physical, psychological and sexual violence, also economic violence. It was recommended that more attention should be paid to the prevention of violence by placing perpetrators on the so-called preventive register and protecting victims by eviction of the aggressors for up to 30 days. The bill was criticized as part of public comment. Large numbers of commentators deemed the proposed actions to be interfering in personal lives of families and expressed outrage that the lawmakers wanted to prohibit parents from raising children with methods which they considered necessary.^32^ The bill, similarly to the one from 2002 and 2018, was not passed. 

The literature on the subject points to the various forms of domestic violence, including physical, psychological, sexual, economic, as well as intentional or unintentional neglect.^33,34^ Sexual violence is very rarely reported because of the embarrassment of the victim and the fear that no one will believe in this type of problem.^35,36^ Hiding this topic can affect young people's awareness in terms of violence. In original research, the vast majority of students from both Poland and Belarus stated that forcing sex on the partner or forcing the partner to watch pornography are not considered as violence.

In the author's original research, almost all students concluded that the use of violence is associated with addiction. These results are identical to those of Zabielska et al., where 78.8% of the studied perpetrators of violence were addicted to alcohol.^37^ Masna et al. also showed a link between the use of violence and addiction in a study where 135 out of 141 perpetrators of violence used drugs.^38^

The results of the study conducted by Giezek et al.^39^ indicate that 26.77% of the 312 reports of domestic violence reported were made within 1 to 3 years of its duration, 13.64% within 4 to 7 years, and in 19.7% of investigated cases - domestic violence was reported or disclosed after as long as 7 years of its duration. These results confirm the opinions of PL and BY students that reporting domestic violence by persons experiencing it may pose an additional threat to the victims on the side of the perpetrator.

## Conclusion

1. In the perceptions of university students re-garding domestic violence in Poland and Belarus, many similarities can be observed regarding the phenomenon itself as well as its causes and manifestations. 

2. Significant differences are probably due to the systemic, legal and cultural conditions of both countries. In Belarus there is more caution in explicit assessment of the phenomena which are regarded as domestic violent in Poland. 

3. The university students of both countries agree that the reason why victims conceal domestic violence is the fear that its disclosure might further worsen their situation. 

4. It is justifiable to constantly raise students' awareness of the mechanisms of domestic violence, its prevention and assistance for the victims.

**Limitations**

In this paper, certain restrictions were found that may have influenced the analysis related to learning the opinions of students on the subject of violence. The analysis was based on a self-made questionnaire, and the results are the subjective feelings of the respondents. For this reason, no unambiguous conclusions were drawn, but on the basis of the acquired results, directions for further actions were indicated. Another limitation refers to the studied sample. The research focused on students of the Faculties of Health Sciences, which does not allow authors to formulate more general conclusions. However, the obtained results indicate the need for further research among students of other faculties and universities.
